# Recurrent cardiac lymphoma: cardiovascular magnetic resonance as a diagnostic key

**DOI:** 10.1093/ehjcr/ytaf611

**Published:** 2025-11-23

**Authors:** Ana Margarida Martins, Catarina Gregório, Joana Rigueira, Ana G Almeida

**Affiliations:** Department of Cardiology, Hospital de Santa Maria (ULSSM), CAML, CCUL@RISE, Faculdade de Medicina, Universidade de Lisboa, Av. Prof. Egas Moniz MB, Lisbon 1649-028, Portugal; Department of Cardiology, Hospital de Santa Maria (ULSSM), CAML, CCUL@RISE, Faculdade de Medicina, Universidade de Lisboa, Av. Prof. Egas Moniz MB, Lisbon 1649-028, Portugal; Department of Cardiology, Hospital de Santa Maria (ULSSM), CAML, CCUL@RISE, Faculdade de Medicina, Universidade de Lisboa, Av. Prof. Egas Moniz MB, Lisbon 1649-028, Portugal; Department of Cardiology, Hospital de Santa Maria (ULSSM), CAML, CCUL@RISE, Faculdade de Medicina, Universidade de Lisboa, Av. Prof. Egas Moniz MB, Lisbon 1649-028, Portugal

## Case description

An 81-year-old woman with a dual-chamber pacemaker and a history of diffuse large B-cell non-Hodgkin lymphoma (DLBCL) was referred to our department for cardiovascular magnetic resonance (CMR) imaging to evaluate an intracardiac mass.

One year earlier, during an admission for acute heart failure, transthoracic echocardiography had identified a large extracardiac mass (65 × 22 mm) in the right atrioventricular groove with right atrial invasion and pericardial effusion (see Supplementary material online, *Figure S1*). Cardiac computed tomography confirmed a mass adjacent to the anterior and inferior walls of the right atrium (see Supplementary material online, *Figure S2*). Endomyocardial biopsy revealed fibrin-associated DLBCL, and the patient completed six cycles of R-CHOP chemotherapy. A post-treatment PET-CT demonstrated residual metabolic activity in thoracic and abdominal lymph nodes.

**Figure 1:**
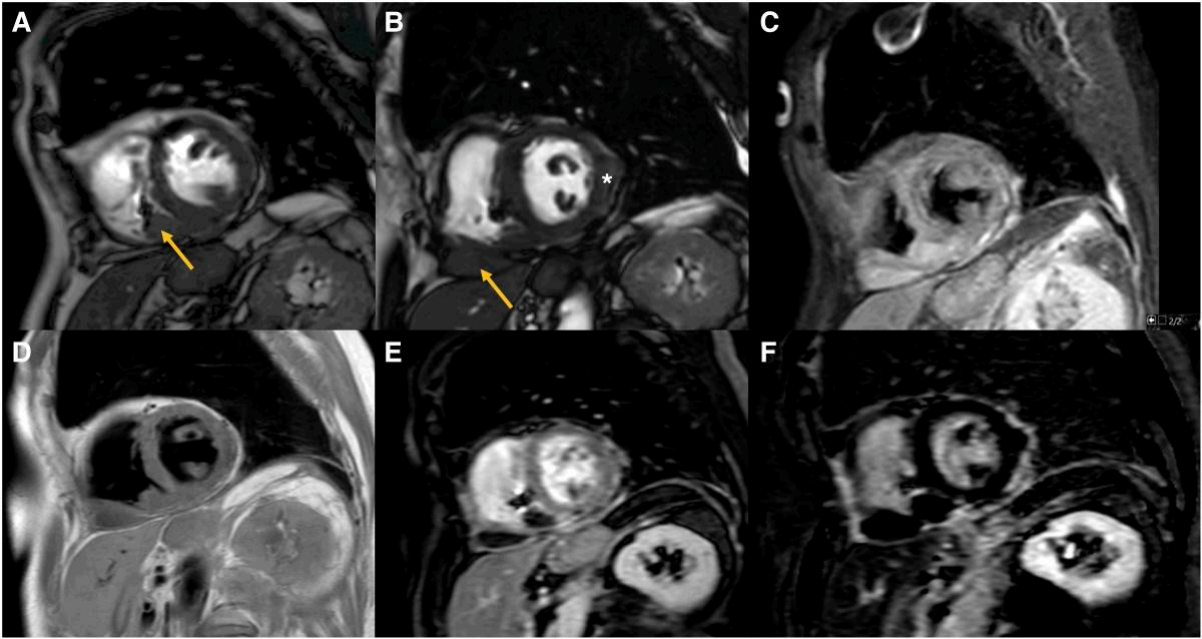
Cardiac mass imaging by cardiac magnetic resonance demonstrated across multiple sequences. (*A* and *B*) Short-axis cine images (balanced Steady-State Free Precession) showing a large mass involving the diaphragmatic wall of the right ventricle (yellow arrow), along with a smaller mass located in the mid-lateral wall of the left ventricle (asterisk). (*C*) T2-weighted sequence showing hyperintensity of the masses. (*D*) T1-weighted sequence showing isointensity of the masses relative to myocardium. (*E*) Early gadolinium enhancement images showing no contrast uptake. (*F*) Late gadolinium enhancement images showing absence of enhancement.

Cardiovascular magnetic resonance was now requested due to recurrent symptoms of anorexia, fatigue, and dyspnoea. Cine imaging identified a 56 × 34 mm mass infiltrating the diaphragmatic wall of the right ventricle and pericardium (*Figure 1A* and *B*; see Supplementary material online, *Figure S3A*), with irregular margins and no cleavage plane. A second, smaller mass was seen in the lateral wall of the left ventricle (*Figure 1B*). The masses were hyperintense on T2-weighted images (*Figure 1C*), isointense on T1 (*Figure 1D*), hypoperfused at rest, and showed no early or late gadolinium enhancement (*Figure 1E* and *F*; see Supplementary material online, *Figure S3B*)—findings consistent with malignancy and suggestive of lymphoma recurrence.

This case highlights the value of CMR in the evaluation of cardiac masses. Compared to echocardiography, CMR provides a wider field of view, multiplanar imaging, and superior tissue contrast, allowing precise assessment of mass size, location, and relationship to adjacent structures, including pericardial involvement, and potential extracardiac extension, such as invasion of the great vessels—key factors in surgical planning. Tissue characterization sequences are key in refining the differential diagnosis.^1^ In this case, the presence of irregular borders, pericardial infiltration without a cleavage plane, T2 hyperintensity, and absence of gadolinium enhancement are features typically associated with malignancy. Notably, the lack of contrast uptake is common in cardiac lymphomas, due to their high cellularity and low extracellular matrix, which limits gadolinium distribution.^2^

Additionally, serial CMR can be used to monitor treatment response following surgical resection or chemotherapy, with no concerns about repeated radiation exposure.^1^

Although the patient had a dual-chamber pacemaker, CMR was safely performed. With the advent of CMR-conditional devices and established safety protocols—including device interrogation and programming, continuous monitoring, and post-scan assessment—CMR is now feasible in most patients with cardiac implantable electronic devices.^3^

## Supplementary Material

ytaf611_Supplementary_Data

## Data Availability

The data that support the findings of this study are available from the corresponding author upon reasonable request.

## Supplementary material

**Consent:** The authors confirm that written consent for submission and publication of this case report, including images and associated text, has been obtained from the patient in line with the COPE guidance.

**Funding:** No external funding received.
