# Supplementary material for: Recurrent cardiac lymphoma: cardiovascular magnetic resonance as a diagnostic key
Source: Eur Heart J Case Rep. 2025 Nov 23;9(12):ytaf611. doi: 10.1093/ehjcr/ytaf611 (PMC12671397; doi:10.1093/ehjcr/ytaf611)
Supplement: ytaf611_Supplementary_Data [file ytaf611_supplementary_data.zip › Clinical case_EHJCR_revista_limpa_1.docx]

**Supplementary material**

**Figure 1** – Transthoracic echocardiography illustrating a predominantly extracardiac mass with myocardial and right atrial invasion.

Figure 1A and 1B - Apical four-chamber views showing a mass extending into the right atrium (blue arrow).

Figure 1C and 1D - Subcostal views demonstrating a large extracardiac mass (65 × 22 mm, blue arrow) located in the right atrioventricular groove with invasion of the right atrium. A large circumferential pericardial effusion is also evident.

**Figure 2** – Thoracic computed tomography (CT) with and without intravenous contrast, demonstrating an intracardiac mass within the right atrium (arrow).

Figure 2A - Non-contrast axial CT showing an isodense mass (blue arrow) relative to the myocardium, located along the anterior and inferior walls of the right atrium. No calcifications are observed. A right-sided dual-chamber pacemaker lead is visible.

Figure 2B - Contrast-enhanced axial CT revealing no enhancement of the mass, consistent with low vascularity. A large circumferential pericardial effusion is also present, measuring up to 30 mm in maximum thickness.

**Figure 3** – CMR imaging showing a cardiac mass involving the right ventricle.

Figure 3A - Two-chamber cine image of the right ventricle demonstrating mass infiltration of the diaphragmatic wall (yellow arrow).

Figure 3B - Two-chamber LGE image of the right ventricle showing absence of enhancement in the right ventricular mass.
