# Supplementary figures and images for: Recurrent cardiac lymphoma: cardiovascular magnetic resonance as a diagnostic key
Source: Eur Heart J Case Rep. 2025 Nov 23;9(12):ytaf611. doi: 10.1093/ehjcr/ytaf611 (PMC12671397; doi:10.1093/ehjcr/ytaf611)

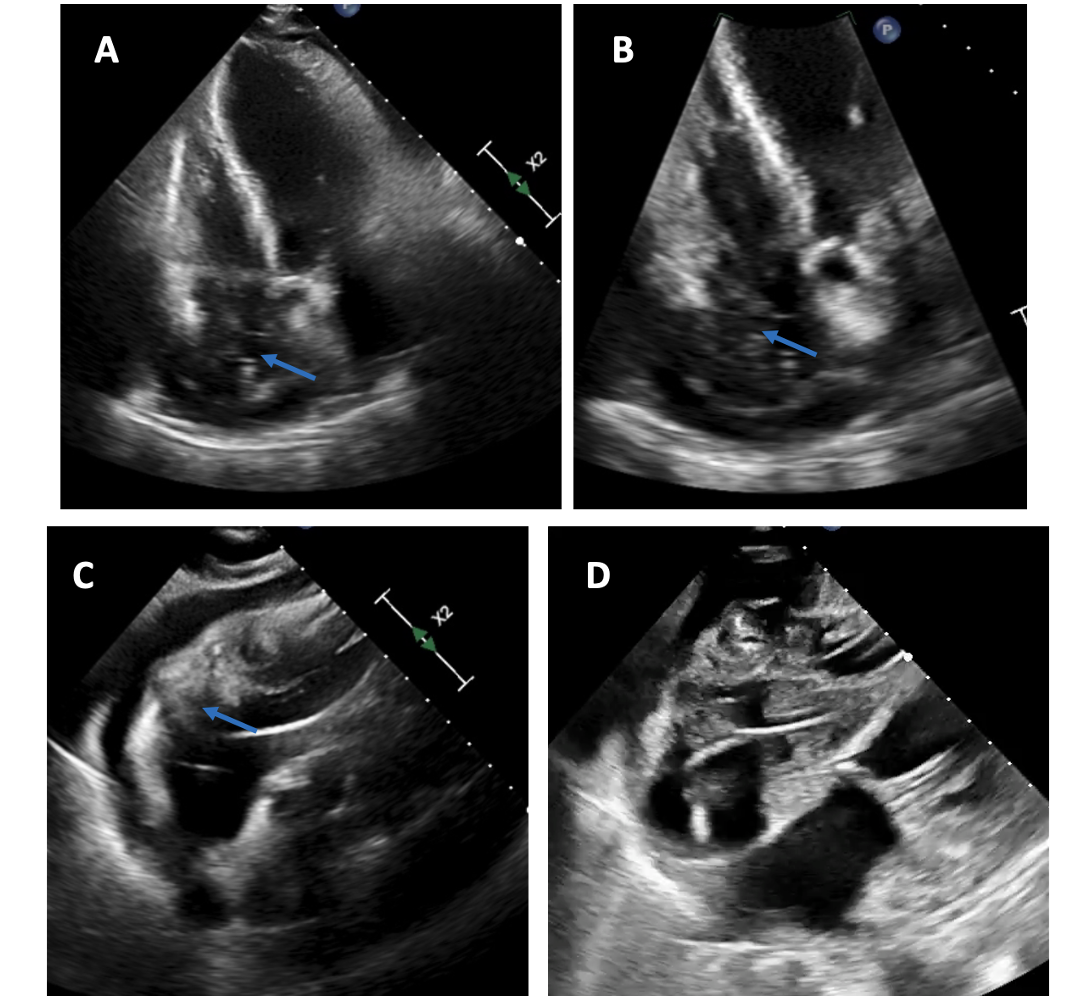

Supplement: ytaf611_Supplementary_Data [file ytaf611_supplementary_data.zip › S_Figure 1.png]

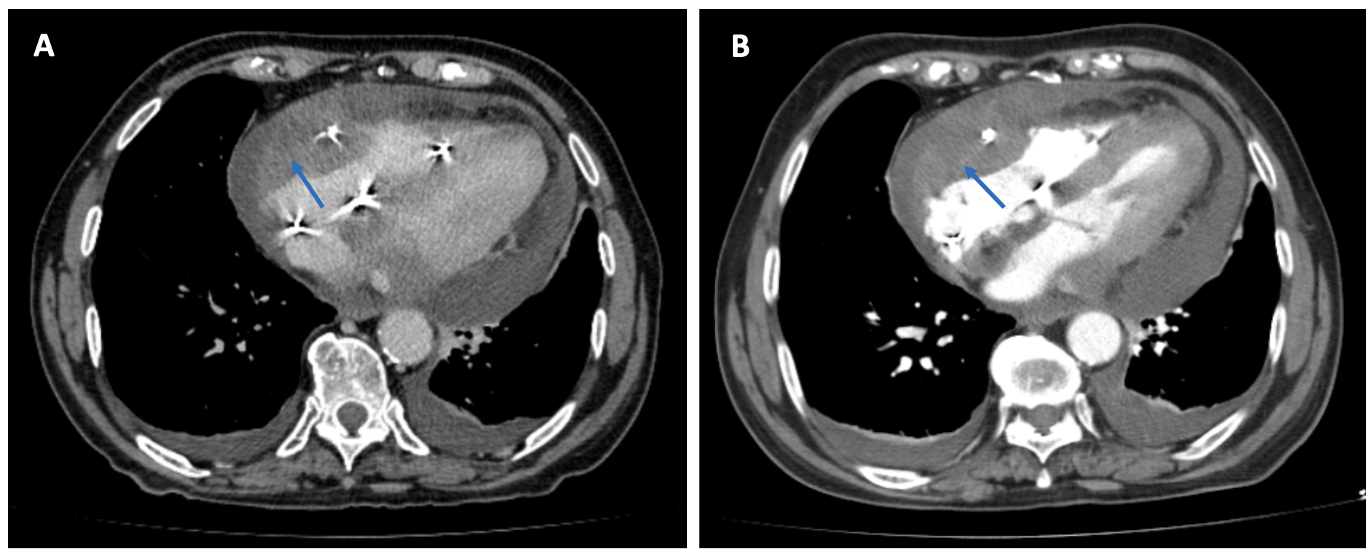

Supplement: ytaf611_Supplementary_Data [file ytaf611_supplementary_data.zip › S_Figure 2.png]

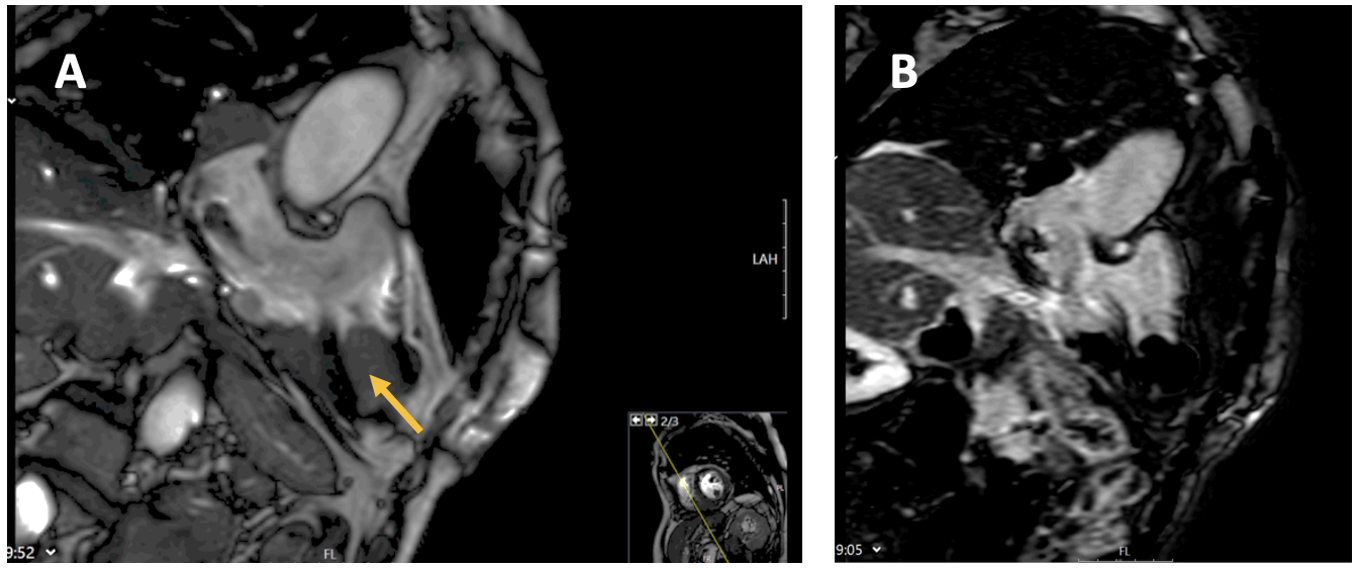

Supplement: ytaf611_Supplementary_Data [file ytaf611_supplementary_data.zip › S_Figure 3.png]
